# Female Human Papillomavirus Infection Associated with Increased Risk of Ectopic Pregnancy: Early Evidence from Taiwan Population-Based Cohort Study

**DOI:** 10.3390/jpm12020172

**Published:** 2022-01-27

**Authors:** Li-Chuan Hsu, Ting-Yu Tu, Hui-Yuan Chen, Renin Chang, Hei-Tung Yip, Mei-Chia Chou, James Cheng-Chung Wei, Kuan-Hao Tsui, Jim Jinn-Chyuan Sheu

**Affiliations:** 1 Institute of Biomedical Sciences, National Sun Yat-sen University, Kaohsiung 804201, Taiwan; taylor6174@gmail.com; 2 Department of Obstetrics and Gynecology, Kaohsiung Veterans General Hospital, Kaohsiung 813414, Taiwan; 3 Department of Orthopedics, Kaohsiung Veterans General Hospital, Kaohsiung 813414, Taiwan; bone200510@gmail.com; 4 Institute of Medicine, Chung Shan Medical University, Taichung 402306, Taiwan; laura812630@gmail.com; 5 Department of Emergency Medicine, Kaohsiung Veterans General Hospital, Kaohsiung 813414, Taiwan; rhapsody1881@gmail.com; 6 Management Office for Health Data, China Medical University Hospital, Taichung 404332, Taiwan; fionyip0i0@gmail.com; 7 College of Medicine, China Medical University, Taichung 406040, Taiwan; 8 Institute of Public Health, National Yangming University, Taipei 112304, Taiwan; 9 Department of Recreation and Sports Management, Tajen University, Pingtung 907101, Taiwan; 10 Department of Physical Therapy, Shu-Zen Junior College of Medicine and Management, Kaohsiung 821004, Taiwan; 11 Department of Physical Medicine and Rehabilitation, Kaohsiung Veterans General Hospital, Pingtung 912012, Taiwan; 12 Graduate Institute of Bioresources, National Pingtung University of Science and Technology, Pingtung 912301, Taiwan; 13 Division of Allergy, Immunology and Rheumatology, Chung Shan Medical University Hospital, Taichung 402306, Taiwan; 14 Graduate Institute of Integrated Medicine, China Medical University, Taichung 404333, Taiwan

**Keywords:** human papillomavirus, ectopic pregnancy, infection, cohort study

## Abstract

Background: This is an investigation of the human papillomavirus (HPV) infection and its correlation with the risk of ectopic pregnancy (EP). Methods: The cohort study includes 11,239 patients with newly diagnosed HPV infections between 2000 and 2012, and by using computer-generated random numbers, patients who do not have HPV infections are selected randomly as the comparison cohort. The HPV infection cohort is matched to comparison individuals at a 1:10 ratio by age and index year. All individuals included in the study were followed up to the point they developed EP, pulled-out from the insurance program, lost to follow-up, or until the end of 2013. A Cox proportional-hazards regression analysis was used to analyze the risk of EP with the hazard ratios (HRs) and 95% confidence intervals (CIs) between the HPV and control cohort. Results: The adjusted hazard ratio (aHR) of EP for HPV patients relative to controls is 1.70 (95% CI = 1.04, 2.78), indicating a positive correlation between EP and HPV in the 13-year follow-up period, after adjusting for age and relevant comorbidities. The sensitivity analyses yield similar results. Conclusions: A history of HPV infection is a potential risk factor associated with the development of subsequent EP in Taiwanese individuals, especially those diagnosed with an HPV infection within 3 years.

## 1. Introduction

The main cause of maternal morbidity in the first trimester is ectopic pregnancy (EP), which leads to about 9% of maternal deaths with an incidence rate of around 10–20 out of every 1000 pregnancies [1]. EP is known as a complication of early pregnancy in which the developing blastocyst becomes implanted outside the endometrium of the uterine cavity, with a 95% incidence rate taking place in the fallopian tubes [2]. Aside from causing embryo premature implantation, chronic inflammation of the fallopian tube resulting in the delayed passage of the fertilized oocyte into the uterine cavity presumably plays the main role in the pathophysiology of tubal pregnancy [3]. Various risk factors account for EP [4,5], for instance, previous EP, pelvic infection or use of intrauterine contraceptive devices (IUD), history of infertility, pelvic inflammatory disease (PID), etc. [4,6,7].

Studies have shown that pelvic infection due to sexually transmitted diseases is associated with a higher risk of developing EP, such as *Chlamydia trachomatis* (odds ratio (OR) 3.07; 95% CI: 1.3–12.3; *p* = 0.002), *Mycoplasma genitalium* (OR 2.3; 95% CI: 1.1–8.6; *p* = 0.03), and the herpes simplex virus (HSV)-1/2 (OR 1.7; 95% CI: 0.75–5.7; *p* = 0.004) [8], or possibly *Neisseria gonorrhoeae* infection [9]. Pelvic infection may change the tubal function and result in pelvic adhesive disease and tubal obstruction.

The most commonly diagnosed sexually transmitted disease is human papillomavirus (HPV) infection [10], which results in more than 6,000,000 new cases every year in America [11]. HPV infections are considered the main cause of anogenital warts [12], and are highly related to the infection-related precancerous and cancerous lesions of the cervix uteri, vulva, vagina, anus, oropharynx, and penis [13,14,15,16,17]. The study reported that patients with PID apparently had a higher risk of cervical cancer and found that HPV prevalence was higher (33.74%) in patients with PID and lower (26.40%) in those without PID (*p* < 0.001) [18]. Patients with a history of PID, on the other hand, appear to have an approximately three-fold increased risk of EP [19]. Upon continual infection, HPV gives rise to the secretion of inflammatory cytokines, which cause immune cell infiltration. The host immune responses and releases of inflammatory cytokines [20] may change the milieu in the upper genital tract that may be related to EP.

Due to no previous studies on the epidemiological relationship between HPV infections and the subsequent development of EP, we carried out this original longitudinal cohort study to explore this important issue and to clarify the association between HPV infection and EP.

## 2. Materials and Methods

### 2.1. Data Source

The data source for our study was the Longitudinal Health Insurance Research Database (LHIRD), a subset of data included in the National Health Insurance Research Database (NHIRD). NHIRD data covers more than 99% of the Taiwanese population (about 23 million people). LHIRD includes data on one million individuals, representing approximately 4% of Taiwan’s population. Between 1996 and 2013, the database had demographic data, inpatient and outpatient care, clinic visits, and information on hospitalization dates and prescriptions. In Taiwan, licensed medical records technicians employed by hospitals verified the coding before claiming the reimbursements, and the National Health Insurance (NHI) Administration authority verified the audit. Nevertheless, data after 2013 were not included because of copyright issues. The authority changed the original identification numbers with surrogate numbers before the data were disclosed to protect the people’s privacy. The Institutional Review Board of the China Medical University in Taiwan approved this study (CMUH104-REC2-115 (AR-6)).

### 2.2. Study Population

We picked out female patients who had been diagnosed with HPV infection (ICD-9 codes 079.4, 795.05, 795.09, 795.15, 795.19) from 2000 to 2012 as the HPV group (*n* = 1136) in the LHIRD. We excluded patients with viral/HPV diagnoses not related to the genital tract (ICD-9-CM 078.1, 078.10, 078.12, 078.19, 796.75, 796.79). The index date was determined as the date of HPV infection diagnosis. Patients with a minimum of one inpatient admission or three outpatient visits for HPV were selected. To secure the accuracy of patients’ information during the study period, this analysis excluded patients with missing demographic data (age and sex). Subjects with an age under fifteen or over forty-five years, history of hysterectomy, cancer, or with EP diagnosed before the index date were excluded. The female control group was selected from LHIRD, randomized, and matched at a ratio of 1:10 by age, index date, and comorbidities including endometriosis, polycystic ovary syndrome (PCOS), benign neoplasm of ovary, PID, miscarriage, uterine leiomyoma, myomectomy, Caesarean section, and IUD. All participants had follow-up until the presence of EP, death, or the end of the study (31 December 2013). In the end, 1,136 subjects were contained in the HPV group and 11,360 non-HPV subjects served as the control group.

### 2.3. Main Outcome and Co-Morbidities

The HPV group was tracked from the index date to the first EP event. The end-point of this study was the occurrence of EP (ICD-9-CM code 633). To refine the accuracy of ICD coding, EP was defined as a patient having a record of EP in minimum of three ambulatory visits or at least one admission. To remove potential bias, we modified the demographic variables and relevant co-morbidities including endometriosis (ICD-9-CM code 617), PCOS (ICD-9-CM code 256.4), benign neoplasm of the ovary (ICD-9-CM code 220), PID (ICD-9-CM code 614), miscarriage (ICD-9-CM code 634), uterine leiomyoma (ICD-9-CM code 218), myomectomy (ICD-9-CM procedure code 68), Caesarean section (ICD-9-CM procedure code 74), and IUD (ICD-9-CM code V45.51 & 996.32). We tracked these baseline co-morbidities by examining at least 2 years’ worth of records before the index date of the incident HPV infection diagnosis.

### 2.4. Statistical Analysis

First, we analyzed the demographic characteristics, including the distributions of categorical age, and co-morbidities between the HPV cohort and non-HPV comparison cohort by the chi-square tests. The incidence density of EP per 1000 person-years was calculated in both cohorts. Second, to investigate the possible effect of HPV, a Cox proportional-hazards regression analysis was performed to estimate the hazard ratios (HRs) and 95% confidence intervals (CIs) after adjustment of covariates. The covariates incorporated into the multivariable models contained age, co-morbidity of endometriosis, PCOS, benign neoplasm of the ovary, PID, miscarriage, uterine leiomyoma, myomectomy, Caesarean section, and IUD. Propensity score matching (PSM) was evaluated using logistic regression to minimize the measurable confounders and potential selection bias in terms of previous mentioned variables. Third, the Kaplan–Meier method was used to describe the cumulative incidence of EP in the two cohorts. The difference between the two cohorts was checked by log-rank test. The incidence of EP was estimated by dividing the number of EP events by follow-up person-years for both cohorts. Fourth, to investigate the effects of age, each mentioned co-morbidity, and follow-up time on the incidence of EP among patients with HPV infection, we applied the multivariable Cox regression model adjusted for age and co-morbidities. The HR adjusted for covariates was calculated for female patients; 15–25, 26–35, and 36–45 age groups; and follow-up times of <1 year, 1 to 2 years, 2–3 years, 3–4 years, and 4–5 years. Last, to minimize observational bias, we performed additional 2 sensitivity analyses. For the statistical outcomes, a *p* value of less than 0.05 was considered statistically significant.

In the primary analysis (Model 1), we examined the temporal relationship between HPV exposure (at least three outpatient visits or 1 admission within 1 year) and the risk of developing EP, adjusted for age and relevant comorbidities (including endometriosis, PCOS, benign neoplasm of ovary, PID, miscarriage, uterine myoma, myomectomy, Caesarean section, and IUD) at baseline. In Model 2, the diagnosis of HPV is restricted to be made by the gynecologists. In Model 3, the definition of exposure to HPV is confined to patients with a medical record of HPV infection within two consecutive years.

## 3. Results

**Figure 1:**
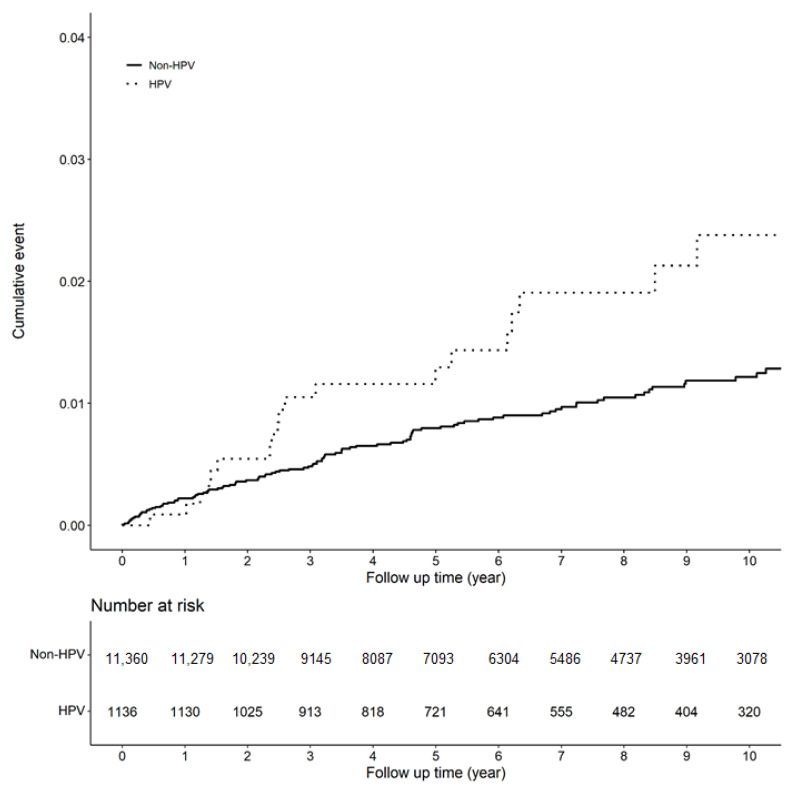
Kaplan–Meier curves of the incidence of EP (ectopic pregnancy).

Figure 1 shows the Kaplan–Meier curves of the incidence of EP in individuals with and without HPV infection.

The baseline characteristics of the patients are listed in Table 1. Our study selected 1136 HPV infection patients and 11,360 non-HPV infection patients with 1:10 matched by age and co-morbidities. The mean ages of the patients in the HPV and non-HPV groups were 34.82 years and 34.89 years in propensity score matching, respectively. Following PS matched, baseline characteristics were balanced.

Table 2 shows that the adjusted hazard ratio (aHR) of EP in HPV patients relative to control patients is 1.70 (95% CI = 1.04, 2.78; *p* = 0.033), indicating a significant difference in these 13-year follow-up periods. Compared to patients aged 15–25 years old, patients aged 26–35 years old showed no significant risk of EP (aHR = 0.85, 95%CI = 0.47, 1.53), and patients aged 36–45 years old had a reduced risk of EP (aHR = 0.17, 95% CI = 0.09, 0.35; *p* < 0.001). The risk of EP was increased in patients with benign neoplasm of the ovary (aHR = 1.65, 95% CI = 1.06, 2.56; *p* = 0.025), in patients with PID (aHR = 2.37, 95% CI = 1.10, 5.12; *p* = 0.028), and in those with myomectomy (aHR = 2.36, 95% CI = 1.06, 5.29; *p* = 0.037).

Table 3 shows the result of the stratification analysis for the association between HPV and EP. In patients of all different age groups (age 15–25, aHR = 0.74, 95%CI = 0.09, 5.98; age 26–35, aHR = 1.92, 95%CI = 1.09, 3.37; age 36–45, aHR = 1.30, 95%CI = 0.39, 4.37), the risk of EP was significantly higher in the age group of 26–35 for HPV patients compared with the matched age group without a history of HPV infection. In the co-morbidity subgroup analysis, HPV infection was found to have a significant impact on the risk of EP in patients without the comorbidities mentioned in Table 1 (aHR = 2.00, 95% CI = 1.01, 3.94; *p* = 0.045).

Table 4 showed the incidence and aHRs of EP stratified by follow-up time. The aHR of EP in HPV group was 5.79 (95% CI = 1.12–30.0; *p* = 0.037), 11.1 (95% CI = 2.16–56.5; *p* = 0.004), 15.6 (95% CI = 3.17–77.0; *p* = 0.001), 5.17 (95% CI = 0.77–34.7; *p* = 0.091), and 1.58 (95% CI = 0.13–19.9; *p* = 0.723) in <1 year, 1 to 2 years, 2–3 years, 3–4 years, and 4–5 years, respectively.

Table 5 provided three models to test the stability of HR of EP in a different definition of HPV exposure (details in Statistical aAnalysis section). We found the aHRs were 1.70 (95% CI = 1.04–2.78; *p* = 0.033), 1.72 (95% CI = 1.05–2.81; *p* = 0.031) and 1.80 (95% CI = 1.01–3.20; *p* = 0.046) in Model 1, 2 and 3. All models yielded a consistent positive association between HPV infection and subsequent EP.

## 4. Discussion

### 4.1. Principle Findings

To the best of our knowledge, this study is the first large-scale cohort study to determine the relationship between HPV infection and the risk of EP. The aHR of EP for HPV patients relative to controls was 1.70 (95% CI = 1.04, 2.78), suggesting a significant positive correlation between EP and HPV in the 13-year follow-up period after adjusting for age and comorbidities. Subgroup analysis suggested that patients within 3 years of the diagnosis of HPV had a significantly higher risk of EP. Compared with the patients aged between 15–25 years, women aged 35 years or older had an aHR of 0.17 (95% CI = 0.09, 0.35) of EP, probably because of lower rates of sexual activity [21] and more prudent contraceptive measures [22], which may result in fewer pregnancies and less EP. The subgroup analysis of our study showed that women with benign neoplasm of the ovary (aHR = 1.65, 95% CI = 1.06, 2.56), PID (aHR = 2.37, 95% CI = 1.10, 5.12), and post-myomectomy (aHR = 2.36, 95% CI = 1.06, 5.29) are at higher risk of EP, which is consistent with another Taiwan NHIRD study done by Hwang et al. [6]. A higher ratio of EP following the diagnosis of a benign ovarian mass may be explained by the EP mass itself, a concurrent corpus luteum, or an ovarian mass that could interfere with tubal function. Post-myomectomy may cause pelvic adhesion that could distort the anatomy and function of the fallopian tube.

### 4.2. Clinical Implications

The potential correlation between HPV infection and EP development remains unclear. More than 95% of EPs are in the fallopian tube ^2^. That happens when a fertilized egg gets trapped on its way to the uterus, often because the fallopian tube is injured by chronic inflammation or with a deformity [3]. On the other hand, in the initial stage of cervical infection, HPV escaped from the immune system without overt inflammation, viremia, or cytolysis, resulting in no activation of the innate immune system. The virus then completes its life cycle in actively dividing cells by employing the host cellular machinery [23]. The following persistent infection triggers the secretion of inflammatory cytokines, which brings about immune cell infiltration. Yet, under immunological surveillance mainly by effector T cells, most HPV infections undergo spontaneous clearance within 1–2 years [24,25]. This possibly explains why the risk of developing EP in HPV-infected patients is higher within the 3-year follow-up periods. Therefore, for HPV-positive women, additional diagnostics with a greater focus on the fallopian tubes could be applied, especially for patients diagnosed with genital HPV in recent years.

### 4.3. Research Implications

Ashshi et al. conducted an M-PCR of seven sexually transmitted organisms in fallopian tubal specimens and found the most prevalent microorganisms detected in EP were *C. trachomatis, M. genitalium,* and HSV-1/2, and they were also associated with a higher risk of EP [8]. Other studies revealed an association between *N. gonorrhoeae* [26], *U. parvum/urealyticum* [27], and *G. vaginalis* [28] with PID and tubal pregnancy. In this retrospective cohort study, we found a significant correlation between HPV infection and EP. Perhaps more attention should be paid to the presence of HPV in fallopian tube tissue and the EP embryo. More research is needed to understand the mechanisms of how HPV infection contributes to EP.

### 4.4. Strengths and Limitations

The strength of this study is an innovative hypothesis relevant to clinical practice and research, and the strength of our research lies in the use of nationwide population-based data and PSM to evaluate the risk of EP in patients with an HPV infection [29]. The advantages of using LHIRD in research include an enormous sample size, population-based data, and long-term comprehensive follow-up. In addition, the negative control outcome was used to minimize residual confounding. Therefore, we think that we have provided ample evidence for this positive association between ectopic pregnancy and HPV symptomatic infection.

However, several limitations inherent to the use of insurance claims databases must be mentioned. First, ICD-9-CM codes for the diagnoses of HPV infection and EP were based on administrative claims data recorded by physicians and hospitals rather than a prospective clinical setting. Inaccuracy may have resulted in misclassification, despite the fact that the bureau of NHI uses an auditing mechanism to minimize diagnostic uncertainty and misclassification. Second, many demographic variables were not provided in the database, such as body mass index, smoking status, lifestyle, socioeconomic status, family medical history, fertility status, the possibility of being influenced by their HPV-infected fertile male partners, and those receiving infertility treatment. We have no information on the cytological results, the corresponding CIN, SIL, or HPV genotyping. These factors would have been valuable for assessing other factors that may be associated with EP. Third, this was a single country evaluation. Fourth, the case numbers of HPV associated EP are relatively small, and our findings may not be suitable for non-Asian ethnic groups. Considering possible ethnic and geographical differences in the incidence and serotypes of HPV, further studies should be conducted in other ethnic groups. Fifth, the HPV vaccination might have altered the immune system of the host, though currently there are no data regarding the HPV vaccination in our NHIRD database because the HPV vaccination is a self-paid service, not covered by NHI, and is therefore not included in the NHIRD.

## 5. Conclusions

In conclusion, this population-based cohort study demonstrated a significant correlation between HPV infection and future ectopic pregnancy in Taiwanese individuals, especially those diagnosed with HPV infection within 3 years. We call for more research to examine the mechanisms by which HPV infection acts to contribute to EP.

## Figures and Tables

**Table 1:** Baseline characteristics of patient with and without HPV.

	Non HPV		HPV		
	*n* = (11,360)		*n* = (1136)		
Variable	*n*	%	*n*	%	*p*-Value
Age, year					0.803
15–25	829	7.30%	82	7.22%	
26–35	4705	41.42%	482	42.43%	
36–45	5826	51.29%	572	50.35%	
mean, (SD)	34.89	(6.46)	34.82	(6.31)	0.727
Comorbidities					
Endometriosis	870	7.66%	89	7.83%	0.832
PCOS	337	2.97%	38	3.35%	0.476
Benign neoplasm of ovary	1478	13.01%	148	13.03%	0.987
PID	344	3.03%	36	3.17%	0.792
Miscarriage	30	0.26%	6	0.53%	0.113
Uterine leiomyoma	1538	13.54%	151	13.29%	0.817
Myomectomy	406	3.57%	37	3.26%	0.582
Caesarean section	55	0.48%	8	0.70%	0.318
IUD	3	0.03%	1	0.09%	0.268

PCOS: polycystic ovary syndrome; PID: pelvic inflammatory disease; IUD: intrauterine contraceptive device.

**Table 2:** Incidence rate and hazard ratio of ectopic pregnancy.

Variables	Ectopic Pregnancy								
	*n*	PY	IR	Crude HR (95%CI)		*p*-Value	Adjusted HR ^†^ (95%CI)		*p*-Value
HPV									
No	105	79,157	1.33	1.00	(reference)	-	1.00	(reference)	-
Yes	19	8006	2.37	1.79	(1.10, 2.92)	0.019	1.70	(1.04, 2.78)	0.033
Age									
15–25	13	4984	2.61	1.00	(reference)	-	1.00	(reference)	-
26–35	86	35,961	2.39	0.94	(0.53, 1.69)	0.842	0.85	(0.47, 1.53)	0.593
36–45	25	46,218	0.54	0.21	(0.11, 0.42)	<0.001	0.17	(0.09, 0.35)	<0.001
Comorbidities									
Endometriosis									
No	112	81,052	1.38	1.00	(reference)	-	1.00	(reference)	-
Yes	12	6111	1.96	1.38	(0.76, 2.51)	0.287	1.27	(0.67, 2.41)	0.461
PCOS									
No	118	85,443	1.38	1.00	(reference)	-	1.00	(reference)	-
Yes	6	1720	3.49	2.27	(1.00, 5.17)	0.051	1.49	(0.64, 3.42)	0.353
Benign neoplasm of ovary									
No	96	76,244	1.26	1.00	(reference)	-	1.00	(reference)	-
Yes	28	10,919	2.56	2.00	(1.31, 3.05)	0.001	1.65	(1.06, 2.56)	0.025
PID									
No	117	84,746	1.38	1.00	(reference)	-	1.00	(reference)	-
Yes	7	2418	2.90	2.07	(0.96, 4.43)	0.062	2.37	(1.10, 5.12)	0.028
Miscarriage									
No	123	87,024	1.41	1.00	(reference)	-	1.00	(reference)	-
Yes	1	139	7.18	4.38	(0.61, 31.4)	0.141	4.07	(0.56, 29.4)	0.165
Uterine leiomyoma									
No	105	75,710	1.39	1.00	(reference)	-	1.00	(reference)	-
Yes	19	11,454	1.66	1.18	(0.72, 1.92)	0.514	1.50	(0.84, 2.68)	0.167
Myomectomy									
No	117	84,570	1.38	1.00	(reference)	-	1.00	(reference)	-
Yes	7	2594	2.70	1.85	(0.86, 3.97)	0.114	2.36	(1.06, 5.29)	0.037
Caesarean section									
No	122	86,644	1.41	1.00	(reference)	-	1.00	(reference)	-
Yes	2	520	3.85	2.82	(0.70, 11.4)	0.146	2.35	(0.58, 9.55)	0.233
IUD									
No	124	87,141	1.42						
Yes	0	22	0.00						

PY: person-years; IR: incidence rate (per 1000 person-years); HR: hazard ratio; PCOS: polycystic ovary syndrome; PID: pelvic inflammatory disease; IUD: intrauterine contraceptive device; †: adjusted by age and all comorbidities in Table 1.

**Table 3:** Stratification analysis.

	HPV											
	No			Yes								
Variables	Events	PY	IR	Events	PY	IR	Crude HR	(95% CI)	*p*-Value	Adjusted HR ^†^	(95% CI)	*p*-Value
Age												
15–25	12	4532	2.65	1	451	2.21	0.84	(0.11, 6.44)	0.865	0.74	(0.09, 5.95)	0.777
26–35	71	32,594	2.18	15	3367	4.45	2.05	(1.17, 3.58)	0.012	1.92	(1.09, 3.37)	0.023
36–45	22	42,031	0.52	3	4188	0.72	1.37	(0.41, 4.58)	0.609	1.30	(0.39, 4.37)	0.674
Comorbidities												
No	50	55,701	0.90	10	5598	1.79	1.99	(1.01, 3.93)	0.047	2.00	(1.01, 3.94)	0.045
Yes	55	23,456	2.34	9	2408	3.74	1.60	(0.79, 3.24)	0.191	1.52	(0.75, 3.10)	0.243

events: number of ectopic pregnancy patient; PY: person-years; IR: incidence rate (per 1000 person-years); HR: hazard ratio; †: adjusted by age and all comorbidities in Table 1.

**Table 4:** The association of the follow-up time and ectopic pregnancy.

	HPV								
Variables	Events	PY	IR	Crude HR	(95% CI)	*p*-Value	Adjusted HR ^†^	(95% CI)	*p*-Value
Duration from last HPV visit to end day									
< 1 year	4	1080	3.70	5.25	(1.16, 23.7)	0.031	5.79	(1.12, 30.0)	0.037
1–2 years	4	610	6.56	9.23	(2.02, 42.2)	0.004	11.1	(2.16, 56.5)	0.004
2–3 years	5	635	4.13	10.7	(1.49, 22.1)	0.014	15.6	(3.17, 77.0)	0.001
3–4 years	2	731	2.73	3.84	(0.63, 23.5)	0.145	5.17	(0.77, 34.7)	0.091
4–5 years	1	70	1.76	2.51	(0.26, 24.3)	0.426	1.58	(0.13, 19.9)	0.723
> 5 years	3	4381	0.68	1.00	(reference)	-	1.00	(reference)	-

events: number of ectopic pregnancy patient; PY: person-years; IR: incidence rate (per 1000 person-years); HR: hazard ratio; †: adjusted by age and all comorbidities in Table 1.

**Table 5:** Sensitivity analyses.

	Compare to Patients without HPV					
	Crude HR	(95% CI)	*p*-Value	Adjusted HR ^†^	(95% CI)	*p*-Value
Model 1: Primary analysis	1.79	(1.10, 2.92)	0.019	1.70	(1.04, 2.78)	0.033
Model 2 ^§^	1.80	(1.11, 2.94)	0.018	1.72	(1.05, 2.81)	0.031
Model 3 ^‡^	1.88	(1.06, 3.35)	0.031	1.80	(1.01, 3.20)	0.046

§: case cohort: woman with HPV infection recorded by gynecologists. Adjusted by age, and all comorbidities in Table 1. ‡: case cohort: HPV patients with diagnosis code for 2 years. †: adjusted by age, and all comorbidities listed in Table 1.

## Data Availability

Data are available from the National Health Insurance Research Database (NHIRD) published by Taiwan National Health Insurance (NHI) Bureau. Due to legal restrictions imposed by the government of Taiwan in relation to the “Personal Information Protection Act”, data cannot be made publicly available. The Longitudinal Health Insurance Database 2000 (LHID2000) was used for this study. There were about 1 million individuals randomly sampled from the Beneficiaries of the National Health Insurance Research Database (NHIRD), that comprised approximately 23.75 million individuals in NHIRD. The detail of LHID2000 please visit the website: https://nhird.nhri.org.tw/en/Data_Subsets.html, accessed on 22 November 2021.
